# Traumatic Tetanus

**Published:** 1864-01

**Authors:** Jos. Jones

**Affiliations:** Surg. P. A. C. S.


CONFEDERATE STATES

JOURNAL.

12257

VOL. I. RICHMOND, JANUARY, 1864. No. 1.

### ORIGINAL COMMUNICATIONS.

ART. I.—*Traumatic Tetanus.* By Prof. JOS. JONES, *Surg. P. A. C. S.*

[In selecting for publication in this number the following extracts from a carefully and elaborately prepared paper now on file in the office of the Surgeon-General of the Confederate States, concerning the nature, history, pathology and treatment of traumatic tetanus, it is due to the author, Surg. Jos. Jones, P. A. C. S., to say that we hope to publish the entire article at some future day; in the meantime, our readers must be content with only such portions as we think have a practical bearing on the treatment of this interesting, but usually fatal malady.—ED.]

*History of the case; Symptoms, &c.*

Confederate General Hospital, Augusta, Ga., July, 1862. Gillstrap, a Confederate soldier; age 37; dark complexion; dark hair and eyes; weight 145 pounds; height five feet six inches; athletic and strong in health; muscles well developed; has been in the service of his country six months; previous to this time his occupation was farming.

At the battle of Secessionville, on James' Island, S. C., June 16th, 1862, whilst standing in a small house, and in the act of taking a cartridge from the box, a Minnie ball passed through a plank in the side of the house, three inches in thickness, and entering the lower part of the anterior fleshy portion of the right fore-arm, passed through the extensor and flexor muscles between the ulna and radius, splintering both bones, without, however, fracturing them across.

The wound suppurated freely, and appeared to be doing well, until July 6th, when the suppuration was sensibly diminished, and the patient complained of spasmodic twitchings and painful sensations in the muscles of the wounded arm and corresponding side, and along the spine, especially between the shoulders and in the region of the cervical and brachial plexus; stiffness and uneasiness about the muscles of the jaws, and painful sense and tightness about the ensiform cartilage; torpor of the bowels, and loss of rest at night. The spasmodic contractions and twitchings of the muscles of the wounded arm were excited and increased by the slightest movements, and were especially aggravated by coughing, which was not only attended with great pain, but also with spasmodic contractions of the muscles of the thorax and neck. For several days previous the bowels have been constipated; and in like manner with this characteristic symptom, the

424816

sense of oppression of breathing, restlessness and loss of sleep, preceded the well marked symptoms of tetanus. The expression of the countenance is that of distress, and even of pain, and the muscles of the face are greatly distorted during the paroxysms.

During the night and following day, (July 7th,) the pain in the arm and between the shoulders and at the præcordium increased, and in like manner the stiffness of the jaws; the tetanic spasms became more violent and frequent; opisthotonos became more marked, and the disturbance of respiration was progressively increased during the paroxysms. Administered in the morning ten grains of calomel, and followed with half a fluid ounce of castor oil in four hours; these purgatives failed to move the bowels. At bed-time administered forty drops of tincture of opium, and as this dose failed to produce sleep, thirty drops more were administered two hours afterwards. After the second dose the patient rested somewhat better, the spasms appeared to be slightly moderated, and he slept a little between the severe spasms.

July 8th, 12 o'clock M.—Says that he feels a little better; just now the spasms do not appear to be so violent; pulse 82, soft and full; respiration quiet and regular between the paroxysms; disturbed and almost entirely arrested during the spasms.

Temperature of hand, 37° 6 C., (99° 7 F.)
" of axilla, 37° 9 C., (100° 3 F)

*Examination of Urine.*—High color, and scanty; amount excreted during sixteen hours, (from July 7th, 8 P. M., to July 8th, 12 M, 4823 grains (Troy;) if from this data the amount of urine be calculated for twenty-four hours, it would equal 7234.32 grains. Specific gravity, 1021.55. Reaction strongly acid; color very deep orange, inclining to red. No deposit after standing more than twenty-four hours; a deposit

| Analysis of Urine, No. 1, July 7th, to July 8th. | Urine passed during 16 hours, July 7th, 8 P. M., to July 8th, 12 M. | Amount and Composition of Urine calculated for 24 hours. | Average Amount and Composition of Urine passed each hour. |
|---|---|---|---|
| | Grains. | Grains. | Grains. |
| Amount of urine | 4823.00 | 7234 32 | 301.43 |
| Water | 4544 26 | 6816.24 | 284.01 |
| Solids | 278 74 | 418.08 | 17.42 |
| Urea | 145.60 | 218.40 | 9.10 |
| Uric acid | 3 73 | 5.52 | 0.23 |
| Free acid | 9 26 | 13 68 | 0.57 |
| Phosphoric acid | 10.89 | 16.32 | 0.68 |
| Sulphuric acid | 7 61 | 11 28 | 0.47 |
| Chlorine | 21.13 | 31.68 | 1.32 |
| Equivalent of chloride of sodium | 34.67 | 51.84 | 2.16 |
| Fixed saline constituents: | | | |
| Entire saline constituents | 67.49 | 101.04 | 4.21 |
| Phosphates of lime and magnesia | 8.76 | 12.96 | 0.54 |
| Sulphates, phosphates and chlorides of alkalies | 58 73 | 88.08 | 3.67 |

fell, however, after standing a longer period, consisting chiefly of the phosphates; a dense white layer, resembling cream in appearance, also rose, and completely coated the surface to the depth of one-eighth of an inch. This was composed of phosphates of lime, magnesia and ammonia, amorphous granules, stellate and prismatic crystals, and vegetable cells. I have seen a similar thick scum rise on the urine of typhoid fever after standing, but never upon the urine of malarial fever.

Upon consultation with my valued and honored friend, Surgeon L. D. Ford, it was determined to try the effects of quinia, and of chloroform and sulphuric ether internally.

Ordered sulphate of quinia; grains v, at 12 M., 2 P. M., 4 P. M., 6 P. M.

Ordered chloroform, drachms iv; sulphuric ether, drachms iv; tincture of opium, drachms iv; mix.

Administered 10 drops of this mixture every hour in sweetened water.

Nine o'clock, P. M., pulse 88; bowels still unmoved. Has taken twenty grains of quinine; does not complain of any unpleasant sensations in head from the action of this. The chloroform mixture has been administered continually each hour, and the patient thinks that it has had the effect of somewhat diminishing the pain and the severity of the spasms. I administered forty drops of laudanum, and ordered this dose to be repeated in two hours; and the chloroform mixture to be continued, ten drops every hour.

July 9th, 12 M.—Continues much the same; pulse 74; skin in a profuse perspiration and feels cool; tongue coated with white fur. The chloroform mixture appears to have slightly diminished the intensity of the spasms; they are, however, very severe and frequent, and his sufferings very great.

Temperature of hand, 37° 55 C., (99° 6 F.)

" of axilla, 37° 85 C., (100° 2 F.)

Continue chloroform mixture; diet, chicken and beef soup, corn gruel and milk punch. His nourishment, as well as his medicine, is taken with great difficulty, and it is only when there is an intermission of the paroxysms, that he is able to suck them carefully through a quill.

Nine o'clock, P. M.—No improvement of symptoms. Continue chloroform mixture; ten drops every hour. Administered now ten grains blue mass; and at 5 A. M., 8 A. M., and 11 A. M., to-morrow morning give five grains quinine; in all fifteen grains.

July 10th, 12 o'clock, M.—Tetanic spasms have increased in frequency and violence; trismus increasing in violence. Bowels obstinately constipated; the blue mass and castor oil have had no effect. Fifteen grains of quinine have been taken this morning without any evident effect.

Nine o'clock, P. M.—No improvement. Administered an enema of molasses, common salt and water; this in like manner with the purgatives, failed to produce any action on the bowels. After the trial with the enema, administered thirty drops of laudanum, and ordered this dose to be repeated in two hours. Ordered to continue chloroform mixture, and in the morning administer five grains of quinine at 5 A. M., 7 A. M. and 11 A. M.

July 11th, 12 M.—Continues to grow worse; bowels still without any movement; has taken fifteen grains of quinine this morning; pulse 84; respiration 15. Ordered chloroform mixture.

*Examination of Urine.*—Light red color. After standing forty-eight hours, notwithstanding the heat of the weather, the reaction was strongly acid. No deposit fell during this time. After standing for a still longer time, however, a deposit of the phosphates was thrown down, and a thick cream like scum formed upon its surface one-fourth of an inch in thickness. The urine emitted a distinct smell of chloroform. Sp. gr. 1024.75.

| Analysis of Urine, No. 4, July 10th, 12 M., to July 11th, 12 M. | Urine passed during 24 hours, from July 10th, 12 M., to July 11th, 12 M. | Average Amount and Composition of Urine passed each hour. |
|---|---|---|
| | Grains. | Grains. |
| Amount of urine | 6457.50 | 269.06 |
| Water | 6185.20 | 253.56 |
| Solids | 372.30 | 15.50 |
| Urea | 302.62 | 12.60 |
| Uric acid | 5.40 | 0.22 |
| Free acid | 21.60 | 0.90 |
| Phosphoric acid | 16.94 | 0.70 |
| Sulphuric acid | 15.56 | 0.64 |
| Chlorine | 10.59 | 0.42 |
| Equivalent of chlorine of sodium | 17.37 | 0.72 |
| Fixed saline constituents: | | |
| Entire saline constituents | Not determined | Not determined. |
| Phosphates of lime and magnesia | 10.39 | 0.42 |
| Sulphates, chlorides and phosphates of alkalies | Not determined. | Not determined. |

Nine o'oclock, P. M.—The tetanic spasms have greatly increased in violence and frequency. Since 2 P. M. emits a shrill, piercing cry during the spasms, and the head and neck are drawn back and downwards towards the heels, whilst the lower extremities are drawn in like manner backwards and upwards towards the head, with great violence. The patient cannot lie down, even in the intermission of the spasms, and is compelled to sit upon the edge of the bed, his lower extremities being forcibly bent backwards under the bed, whilst the neck and head are bent backwards over the bed. The jaws are very rigid; it is impossible even, during the most complete remissions, to protrude the tongue; and it is only by the greatest care, and by a careful selection of the more complete remissions, that he is able to take his chloroform mixture, and a little nourishment. Complains of great pain and "drawing" in the muscles between the shoulders, in the neck, (and in the region of the diaphragm.) The wound in the arm looks dry and bluish and purplish red, and has ceased to discharge pus; the discharge now consisting of a small quantity of serous fluid. Tincture of iodine was poured into the wound, and the fore-arm was also painted with it. The application of the tincture of iodine, directly to the wound, appeared to increase the spasms. Immediately after, the patient had several terrible spasms, during which he emitted most sharp and piercing cries. Has passed no urine since 12 M., and says that he is unable to do so.

I administered thirty drops of laudanum, together with thirty drops of the mixture of chloroform, ether and laudanum, and directed a repetition of this dose in two hours, together with the hourly administration of fifteen drops of the chloroform mixture; and in the morning five grains of quinine at 5 A. M., 7 A. M. and 9 A. M.

July 12th, 12 M.—Spasms not so violent as yesterday; appears to have been benefitted by the increased dose of the chloroform mixture. Pulse, respiration and temperature continue much the same. Pulse 84 per minute; intermittent during the spasms.

Has taken fifteen grains of quinine this morning. Thus far I have observed no beneficial effects whatever from the quinine. On several occasions it has even appeared to aggravate the spasms, whilst on the other hand improvement always appears to follow the free use of the chloroform mixture. Administered thirty drops of laudanum and thirty drops of the chloroform mixture, and repeated this dose half an hour after, and ordered the continuance of fifteen drops of the mixture each hour.

Nine o'clock, P. M.—There appears to be a slight improvement of symptoms; pulse 88. Has passed no urine this day since 12 M. Bowels still obstinately constipated.

Painted the entire arm with tincture of iodine, and poured the tincture into the wound. This caused some pain and an increase of the spasms. Administered forty drops of laudanum, with thirty drops of the chloroform mixture, and ordered this dose to be repeated in two hours, and also the continuance of the mixture; fifteen drops each hour.

July 13th, 11 A. M.—Appears somewhat easier. The second dose of laudanum and chloroform mixture, administered last night, induced some rest, and he slept two hours. The tetanic spasms appear to have lessened a little in severity, and the wound is commencing to suppurate.

Administered thirty drops of laudanum and thirty drops of chloroform mixture, and ordered the chloroform mixture to be continued in the usual dose, and also five grains of quinine at 12 M., 2 P. M. and 4 P. M.

Nine o'clock, P. M.—Has passed more urine than usual this day. His symptoms have changed for the worse—the paroxysms are more violent and the sufferings more acute—cries out in each spasm.

Twenty-five drops of laudanum, with an equal number of the chloroform mixture, were administered; and as they appeared not to afford any special relief, the dose was repeated at the end of half an hour. The continuance of the chloroform mixture as usual, together with five grains of quinine, at 5 A. M., 7 A. M. and 9. A. M., were ordered.

July 14th, 11 A. M.—Did not rest during the night. Has taken fifteen grains of quinine this morning. Ordered Epsom salts, one ounce; water, six ounces; mix and administer at once. Ordered chloroform, gum camphor, laudanum, sweet oil, four drachms each; dissolve the camphor in the chloroform, and then mix with the laudanum and sweet oil. Use this as a liniment to the muscles of the back. Continue the application of tincture of iodine to the wound, and also rub the entire arm with iodine ointment, and apply a plaster of iodine ointment over the wound.

Nine o'clock, P. M.—Through mistake the nurse failed to administer the chloroform mixture, and the patient has suffered much this day. The Epsom salts failed to exert any effect upon the bowels. The chloroform liniment afforded some slight relief of the pain between the shoulders.

Ordered Epsom salts, one ounce; water, six ounces; mix and administer now. Ordered laudanum, m. xl; chloroform mixture, m. xl; sweetened water, one ounce; mix and administer in half an hour. Continue chloroform mixture and liniment.

July 15th, 12 M.—More comfortable; spasms not so violent, and appear to be diminishing both in frequency and force. Pulse 84; regular. Respiration in the remissions regular. Temperature of the extremities and trunk quite uniform, and about the same as last recorded. Says that he obtained some rest last night. Administered thirty drops of laudanum, and the same number of the chloroform mixture, and ordered the mixture continued as before.

July 16th, 10 A. M.—Much better; spasms much less severe; jaws are much more relaxed, and he is able to talk with more ease. Pulse 86; respiration 18. Ordered to paint arm with tincture of iodine, and continue chloroform mixture.

Nine o'clock, P. M.—Pulse 100, in sitting posture, and intermittent after the severe spasms. Twenty-five drops of laudanum and an equal number of chloroform mixture, with one ounce of brandy properly diluted, were administered with good effect; and this dose repeated in half an hour, resulted in the best night's rest since the onset of the disease.

Ordered enema of salt and molasses now, and if without effect, repeat in the morning.

July 17th, 10 A. M.—Continues to improve; rested much better during the night than at any previous time. The spasms continue to diminish in frequency and force. Pulse in sitting posture 82, full, soft and good; tongue coated with light white fur. Continue chloroform mixture.

Nine o'clock, P. M.—Suffering somewhat more this evening. Ordered brandy, two ounces; laudanum, one ounce; chloroform, m. xxx; sweetened water, six ounces; mix; and divide into two parts; one to be taken now, and the other at the end of one hour. Continue the chloroform mixture.

July 18th, 11 A. M.—Much better; says that he feels like a new man; rested well during the night; the expression of his countenance has greatly improved; pulse 80; respiration 14; temperature as in previous observations. Twenty drops of laudanum, with an equal quantity of chloroform, were administered, and this dose was repeated at the end of one hour. Continue chloroform mixture.

(He continued thus steadily to improve, taking regularly the chloroform mixture, and occasionally castor oil, when indicated; which latter, assisted by enemas of molasses, salt and water, happily succeeded in producing on the 21st the first real evacuations from the bowels since the 6th of July.—Ed.)

July 22d, 9 P. M.—Pulse 80; has had a few light spasms, but upon the whole has been quite comfortable. The muscles of the jaw have greatly relaxed as the other symptoms improved, and he can now open his mouth with considerable ease.

Administered thirty drops each of laudanum and chloroform, with one fluid ounce of brandy, properly diluted, and ordered an ounce of salt and molasses for the morning. Continue chloroform mixture.

July 23d, 12 M.—Much better; has had no general spasms, only slight twitchings of the muscles of the arms; rested well during the night; pulse 80. Ordered enema of molasses and salt. Continue chloroform mixture.

9 o'clock, P. M.—Says that he does not feel as well this this morning; pulse 90, full and strong; surface feels a little warmer than usual. The enema this morning produced an evacuation of hard balls.

Ordered Dover's powder, ten grains, now, and repeat in two hours, if rest is not produced. Continue brandy, wineglass full every two or three hours, also the chloroform mixture, and in the morning repeat the enema.

July 24th.—Says that he feels better; having taken both Dover's powders, rested well during the night. The enema has been retained, and has not yet acted; says that he feels an inclination to evacuate the bowels; pulse 76.

Temperature of hand, 37° 6 C., (99° 8 F.)
" of axilla, 38° 4 C., (101° 2 F.)

This is the first morning that the patient has complained of the sensation of hunger. Examined the wound carefully with a probe, and extracted from the orifice upon the internal surface, at which the ball entered, a flat piece of lead about one-fourth of an inch in diameter, the edge of which had come in contact with the radius, and was curled over, having evidently impinged against the bone; for small fragments of bone were impacted in the lead. This particle of lead was most probably detached from the ball during its passage through the thick plank; the remainder of the ball, as before stated, passed entirely through the fore-arm between the radius and the ulna. This fragment of the ball was surrounded by a thick fibrous capsule. The edges and sides of the wound upon both surfaces, the internal and external (points of entrance and exit to the ball), were much thickened and hardened, and the bones along the track of the ball have evidently been injured. Owing to the severe spasms which attempts at even slight examinations induced, a thorough examination of the wound was not made until this time. The examination with the probe was not made when the patient first entered the hospital, for he had been wounded several days, and the wound appeared to be doing as well as usual, in the most favorable cases. I made incisions upon both surfaces through the thickened tissues, and introduced a tent, smeared with basilicon ointment, for near one inch, in the track of the wound; painted the surrounding parts with tincture of iodine, and ordered the fore-arm to be rubbe dwith the following liniment: Chloroform, one ounce; gum camphor, four drachms; laudanum, four drachms; sweet oil, three ounces; dissolve the camphor in the chloroform and mix well with the other ingredients. Has taken no chloroform since 3 A. M. this morning. Ordered the chloroform mixture stopped.

July 25th, 12 M.—Last evening at 8 P. M., says that he had a "very bad turn;" felt as if he was all drawn up and stiff, and unable to breathe; and the muscles of the arm, back and body generally were spasomed. The return of the spasms appeared to be due to the sudden discontinuance of the chloroform mixture. The chloroform mixture was recommenced immediately after the spasm; forty drops of this, with forty drops of laudanum, produced some relief, and he fell asleep about midnight. This day he is quite comfortable; pulse 76. The wound does not look so well; suppuration is somewhat diminished; urine light-colored, with heavy deposit.

Ordered to continue chloroform mixture, fifteen drops every hour, and administer now an enema of salt, molasses and turpentine. The patient has been taking brandy frequently during the attack, averaging about eight ounces each day.

9 o'clock P. M.—The chloroform mixture has produced good results.

Ordered calomel, grains xii; follow with castor oil, one ounce, in the morning. Continue the brandy and chloroform mixture.

July 26th, 12 M.—The calomel and oil produced the desired effect, and are still acting; says that he feels much better. The daily introduction of a tent, smeared with basilicon ointment, into the wound, has produced good results, causing a profuse suppuration, and the evacuation of a deep-seated abscess between the wound and the elbow joint. This morning this deep-seated abscess discharged suddenly through the wound several fluid ounces of pus, to the great relief of the patient. After this the swollen parts of the fore-arm below the elbow were sensibly reduced in size.

Nine o'clock, P. M.—Has been taking only ten drops of chloroform mixture each hour during the day, and has intermitted several times, so that not more than sixty drops have been administered. The pains and contractions and spasms of the muscles of the arm and legs and back have commenced again; the diminution of the chloroform has been attended by a return of the paroxysms. The symptoms, however, are very mild, and for the first time since his attack he has been able to stand on his feet, and to walk across the ward, supported on either side by assistants. Pulse 76. The purgative last administered has acted nine times. Thirty drops each of laudanum and chloroform, with one fluid ounce of brandy, were administered, and the chloroform mixture ordered regularly—ten drops each hour.

July 27th, 10 A. M.—Rested well after midnight; all his symptoms better; pulse 76; urine light colored.

Ordered to continue chloroform mixture, ten drops each hour.

July 28th, 12 M.—Continues to improve; the swelling in the arm has almost entirely subsided; says that he feels quite well, with the exception of stiffness in the muscles and some pain between the shoulders, and is hungry all the time. Pulse 84; bowels readily acted on by the medicine.

P. M.—Continues quite comfortable; complains only of a slight cough and pain in the muscles of the back—most probably the result of the severe tension of the muscles during the previous spasms.

July 29th.—Continues to improve and walks about the ward.

July 30th.—Continues to gain strength; wound looks well and is rapidly healing. Amount of urine excreted from July 28th, 12 M., to July 30th, 12 M., (forty-eight hours,) grains, 51206.70; specific gravity, 1016. Natural yellow color.

P. M.—Has taken no chloroform this day; nothing but brandy. Has experienced no ill effects from the change. Temperature of axilla, 40° 2 C., (104° 4 F.)

July 31st.—Walks about; says that he feels perfectly well; tongue clean; urine normal in appearance; has taken no chloroform during the last two days; takes nothing but a tablespoonful of brandy every three or four hours.

August 1st, 12 M.—Appears to be entirely restored. The improvement of the arm has been very rapid; the symptoms of irritation, inflammation and swelling have almost entirely disappeared, and the wound is nearly closed. Takes no medicine except a little brandy punch occasionally. Bowels moved regularly each day; pulse 84; respiration 20. The intermittent action of the pulse has disappeared entirely with the disappearance of the spasms. Urine normal in color; specific gravity, 1013.

| Analysis of Urine, No. 17, passed July 31st, 12 M., to August 1st, 12 M., (24 hours.) | Amount and Constituents of Urine passed during 24 hours, July 31st, 12 M., to August 1st, 12 M. | Average Amount and Composition of Urine passed each hour. |
|---|---|---|
| | Grains. | Grains. |
| Amount of urine | 28060.10 | 1169.07 |
| Water | 27190.53 | 1132.84 |
| Solids | 869.57 | 36.23 |
| Urea | 543.48 | 22 64 |
| Uric acid | 11.46 | 0.47 |
| Free acid | ......... | ......... |
| Phosphoric acid | 15.98 | 0.66 |
| Sulphuric acid | 20 62 | 0.85 |
| { Chlorine | 120.30 | 5.01 |
| { Equivalent of chloride of sodium | 197.47 | 8.22 |
| Fixed saline constituents: | | |
| Entire saline constituents | 296 72 | 12.34 |
| Phosphates of lime and magnesia | 18.64 | 0.77 |
| Sulphates, phosphates and chlorides of alkalies | 278.08 | 11.58 |

This patient continues to gain strength; the wound healed up; and he was sent home on a furlough ten days after this observation.

